# Effect of age, sex, height, ethnicity, and femoral bowing on the anatomical fitting of the LCP distal femur plate

**DOI:** 10.1007/s00402-025-06079-1

**Published:** 2025-10-14

**Authors:** Beat Schmutz, Minh Tri Phan, Jeremy Pople, Bertha Ching Wai Lam, Eden Schoofs, Jacelle Warren, Jaimi Conlon, Hiroaki Minehara, Kevin Tetsworth, Michael Schuetz

**Affiliations:** 1 Queensland University of Technology, Brisbane, Australia; 2 Jamieson Trauma Institute, Brisbane, Australia; 3 Hospital for Traumatology and Orthopaedics, Ho Chi Minh, Viet Nam; 4 University of Queensland, Brisbane, Australia; 5 Fukushima Medical University, Fukushima, Japan; 6 Royal Brisbane and Women’s Hospital, Brisbane, Australia

**Keywords:** Distal femur, Fracture, Pre-contoured plate, Plate fit, 3D model, Anterior bow

## Abstract

**Purpose:**

Lateral locking-plate fixation is commonly used for distal femur fractures. Pre-contoured plates attempt to match the bony anatomy of the target patient population. However, plate fit is highly variable due to inter-subject morphological differences. If plate misfit is not recognised and addressed, axial malalignment may arise. This study evaluates the effects of age, sex, height, ethnicity, and femoral bowing on the anatomical fit of a distal femur plate.

**Methods:**

Unilateral 3D models of 80 (40 male, 40 female) Caucasian and 79 (34 male, 45 female) Vietnamese femora were utilised. Both cohorts consisted of young (< 65 years) and old (≥ 65 years) subjects, with 40 young and 40 old Caucasian, and 36 young and 43 old Vietnamese. The plate undersurfaces of 9-, 11- and 13-hole LCP Distal Femur plate 3D models were positioned on bone models and anatomical fitting was assessed through application of developed clinical criteria.

**Results:**

Satisfactory plate conformity was achieved from plate head up to hole 6, with most measurement locations fitting 52–100% of bones from both ethnicities. There was tendency towards proximal plate misfit from hole 8 onwards with 0–41% fit achieved in this region, and mean distances of 11.6 mm and 16.3 mm being observed at the proximal plate tip for Caucasians and Vietnamese respectively (*p* ≤ 0.017). Patient sex, height, ethnicity, and femoral bowing all had significant impacts on fit, while the effect of age was limited. Sex and ethnicity related height differences would suggest that patient height and femoral bowing are the main variables affecting fit.

**Conclusions:**

The observed proximal plate misfit for both Caucasians and Vietnamese suggests LCP Distal Femur plates may exhibit less anatomical conformity than generally assumed. Where plates are used as a reduction tool, pre-operative templating of the intact contralateral femur may help identify plate misfit.

**Supplementary Information:**

The online version contains supplementary material available at 10.1007/s00402-025-06079-1.

## Introduction

 Lateral locking plate fixation is a common treatment for distal femur fractures. Commercial anatomically pre-contoured plates attempt to closely match the bone contour of the lateral distal femur and the femoral shaft for the majority of the target patient population. Due to their pre-contoured shape they often require no or minimal intraoperative shaping [15]. For comminuted fractures in particular, they also provide a template for anatomical reduction of fragments [3, 32]. Furthermore, these plates reduce the incidence of implant prominence, resulting in less irritation of surrounding soft tissue, particularly in the lateral condylar metaphyseal region.

Biomechanically, the current use of locking screw technology to stabilise the implant against the bone negates the need for a perfect fit between plate undersurface and bone [8]. This has allowed for the development of a generalised implant shape [9]. While a perfect fit is not required, particularly in the muscle covered diaphysis, the efficacy of the construct can be compromised by increasing plate to bone distances, as this increases the bending moment and loading on the locking screws [1]. Therefore, a compromise must be found between a shape that generates clinically acceptable gaps between bone and implant, for the majority of the target patient population, and one which ensures mechanical stability for fixation. Long documented diversity in bone morphology between varying demographics, such as age, sex and ethnicity have, and continue to, challenge the process of designing fracture fixation plates [34].

Despite generally good results, anecdotal clinical feedback, confirmed by a clinical study on Asian cadaver bones, suggests there is tendency for significant proximal misfit when using anatomical pre-contoured LCP Distal Femur (Depuy Synthes) locking plates [15]. Applications of 11-hole plates have demonstrated adequate alignment along the lateral femoral shaft, with the exception of the proximal area, where misalignment resulted in an average bone-proximal plate tip gap of 11 mm (range 0–36 mm) [15]. Even though the bone in this area is surrounded by a considerable muscle and soft tissue envelope, a >20 mm gap (seen in 10% of specimens) between bone and implant was seen as clinically unacceptable [15]. When surgeons attempt to reduce gaps in these instances, through pulling the bone closer to the implant, valgus malalignment of the femoral fracture arises, which has the potential to lead to undesirable changes in load distribution across the knee post-surgery [15].

More recent studies of different commercial distal femoral pre-contoured locking plates, in relation to total knee arthroplasty (TKA), demonstrate under-contouring of plates and a tendency toward plate misfit in femur metaphyseal regions [2, 5]. Within this region, average plate to bone gaps have been found to range from 6 to 9 mm pre-TKA, with gaps being more significant post TKA in one study [5] but not the other [2]. Sex differences have also been observed, with females exhibiting smaller gaps pre-TKA and larger gaps post-TKA [5]. Post-operative recovery in the setting of significant gaps may result in medialisation of the distal fragment, causing golf club deformities or, if not recognised, valgus malalignment of distal segments [2, 5].

Despite the various plate fit studies conducted over the last two decades [10, 14, 17, 19, 23, 28, 32, 33], to our knowledge, the extent to which the reported proximal plate misfit for Asian bones also impacts Caucasian femora remains unknown. Similarly, while metaphyseal plate misfit has been reported for predominantly Caucasian patients and observed clinically in Asian patients, this misfit has not been quantified in a single study utilising a multi-ethnic bone dataset. While Hwang et al. [15] observed that the amount of misfit was related to the extent of the lateral bow of the femur in the coronal plane, the effect of anterior femoral bowing on plate fit has not yet been assessed. Pre-contoured femoral plates have an anterior bow radius that attempts to match the typical femoral bow radius. However, previous studies have demonstrated a positive correlation between patient height and femoral bow radius of curvature (ROC), as well as age, sex, and ethnicity differences [6, 12, 16, 20, 21, 27, 30]. It is therefore reasonable to assume that a mismatch between plate and bone bow radius would also affect plate fit.

Based on available knowledge, our hypothesis was that, at least in the proximal shaft, the plate would fit better to the Caucasian anatomy than the Vietnamese. Therefore, the principal aim of this study was to determine the effect of differing demographic parameters, such as age, sex, height, and ethnicity, along with femoral anterior bow, on the anatomical fit of a pre-contoured distal femur plate, in order to better inform the clinical community and aid future implant shape optimisation. A protocol for quantitative anatomical plate fit assessment was first developed based on clinical requirements. The protocol was then applied to quantify and analyse plate fit for an age diverse multi-ethnic set of 3D bone models.

## Materials and methods

### 3D bone models

Unilateral 3D bone models (outer and inner bone cortex surface) of 80 (40 male, 40 female) Caucasian and 79 (34 male, 45 female) Vietnamese femora were utilised from previous studies (unpublished). Both cohorts were separated into subgroups of young (< 65 years) and old (≥ 65 years) subjects, with groups of 40 young and 40 old Caucasian, and 36 young and 43 old Vietnamese established.

The 3D bone models of young Caucasians were generated from MRI scans of volunteers at the Princess Alexandra Hospital, Brisbane, Australia (PAH HREC Approval: 2007/176), utilising multi-threshold segmentation combined with manual segmentation [25, 26]. The bone models of old Caucasians were reconstructed from postmortem CT images acquired of specimens from Queensland University of Technology’s body bequeath program (QUT UHREC Approval: 2000001080). All Vietnamese bone models were generated from non-identifiable clinical CT images retrospectively retrieved from Medic Medical Centre, Ho Chi Minh, Vietnam (QUT UHREC Exemption: 1600001161). Both CT and MRI based 3D models were generated using Amira 2020.2 (Thermo Fisher Scientific, Waltham, MA, US). All bone models were visually inspected and considered to be of normal appearance.

### 3D plate models

Digital 3D models of a 9-hole, 11-hole, and 13-hole LCP Distal Femur plate (DePuy Synthes, Switzerland) were used. A 11-hole and a 13-hole plate were 3D laser scanned (Solutionix Rexcan CS optical scanner, Solutionix, Seoul, South Korea). The plates were re-scanned with inserted locking screws for correctly determining screw trajectories. Utilising reverse engineering tools in Rapidform2006 (Inus Technology, Korea), the plate undersurface and screw trajectories were obtained from the 3D laser scans. The 9-hole plate undersurface was generated by shortening the 11-hole plate. For each bone, the longest plate length was selected such that the proximal tip was ≥ 2 cm away from the base of the lesser trochanter. The longest clinically suitable plate for each bone was chosen as this represents the worst-case scenario in terms of anatomically fitting a plate to a bone. In addition, this avoids bias which would likely result from using a fixed length plate, based on the shortest bone in the dataset, as this is expected to fit better for Caucasians and males since they have longer bones on average compared to Asians and females.

### Plate positioning

**Fig. 1:**
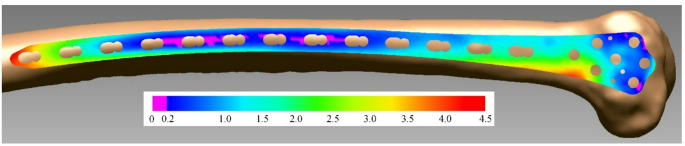
Correct surgical alignment of plate undersurface, with at least three areas of plate-bone distance ≤ 0.2 mm (pink regions). The plate is represented by the colour-heat map of the deviations between plate and bone

Through an iterative process using reverse engineering software (Rapidform2006, Inus Technology, Korea), the undersurface of the plate was positioned correctly on the bone model (Fig. 1).

The undersurface was positioned to achieve plate-bone contact (plate-bone distance ≤ 0.2 mm) at a minimum of three locations: distal anterior, distal posterior, and shaft, analogous to Hwang et al. [15]. The collision detection tool was used to ensure that at no point was the plate undersurface penetrating the bone surface. Further, it was ensured that there was no screw penetration in the intercondylar fossa, and the most proximal screw was located within the intramedullary canal.

### Definitions of plate fit and quantification of fit

**Fig. 2:**
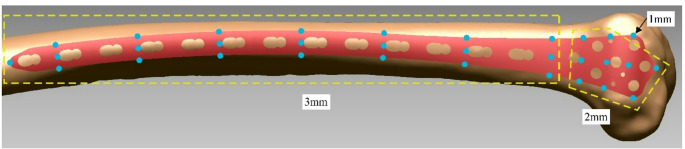
13-Hole plate undersurface positioned on lateral distal femur with fit criteria for shaft and plate head. Light blue dots show locations of the plate-bone distance measurements, along with maximal acceptable distance thresholds

An anatomical fit of the plate was defined as one satisfying three criteria which were defined in an effort to satisfy both clinical and biomechanical requirements, while leaving spatial freedom to accommodate for morphological differences of bones in the populations of interest. We utilised distance as metric [28] for quantifying plate fit rather than volume [10, 32], as the latter on its own does not provide any information regarding maximum or average distance between plate and bone for a particular region of interest [28], i.e. it does not provide information on plate prominence/protrusion. The fit criteria (Fig. 2) were defined as:

- Criteria 1: Plate to bone distance ≤ 1 mm at most anterior distal location;
- Criteria 2: Plate to bone distance ≤ 2 mm at nine points over the metaphyseal region, including distal tip;
- Criteria 3: Plate to bone distance ≤ 3 mm for points (anterior, centre, posterior) along the shaft spaced at ~ 4 cm intervals, including proximal tip.

A customised batch-process module (MATLAB, MathWorks, USA) was developed to measure and record the plate-bone distances of the plate-bone assemblies. The MATLAB script measured the Euclidean distances from the point of interest on the plate to each vertex on the bone model. The shortest distance was selected and recorded. The accuracy of the custom script was validated by verifying that the shortest distance measurement matched the one obtained with the reverse engineering software Rapidform2006. Overall anatomical plate fit was defined as satisfying all three criteria.

### Femoral anterior bow radius and plate radius measurements

The femoral radius of curvature (ROC) was measured to assess the effect of mismatch to plate bowing radius on plate fit. In Rapidform2006, the radius of the 3D canal antecurvature was determined from a best-fit circle to the centre points of 20 axial cross-section curves, equally spaced along the diaphysis of the inner bone cortex model. The level of the proximal diaphysis was defined by the base of the lesser trochanter, whereas the distal end was defined at ~ 10 mm proximal to the start of the lateral condyle, respectively [27]. The ROC measurements were available from previous studies (unpublished). The plate ROC was determined in Rapidform2006 from the best-fit circle to the centre curve of the shaft part of the reverse engineered plate undersurface.

### Statistical methods

In a first step, descriptive statistics for continuous and categorised data were produced. Due to non-normal distributions of several measurements, the Mann-Whitney U test for two independent samples was used to test for significant differences between patient age (young vs. old), sex (male vs. female), ethnicity (Caucasian vs. Vietnamese) and the effects of age, sex and ethnicity on plate fit (median plate-bone distances). The Chi-Square test was used for comparing the difference in satisfying plate fit criteria between two groups. The associations between age, height, ROC, and plate fit were explored using nonparametric correlation analyses following Spearman. The collated data were statistically analysed using SPSS version 29.0 (IBM Corp., Armonk, NY) with the level of statistical significance set to *p* < 0.05.

## Results

**Fig. 3:**
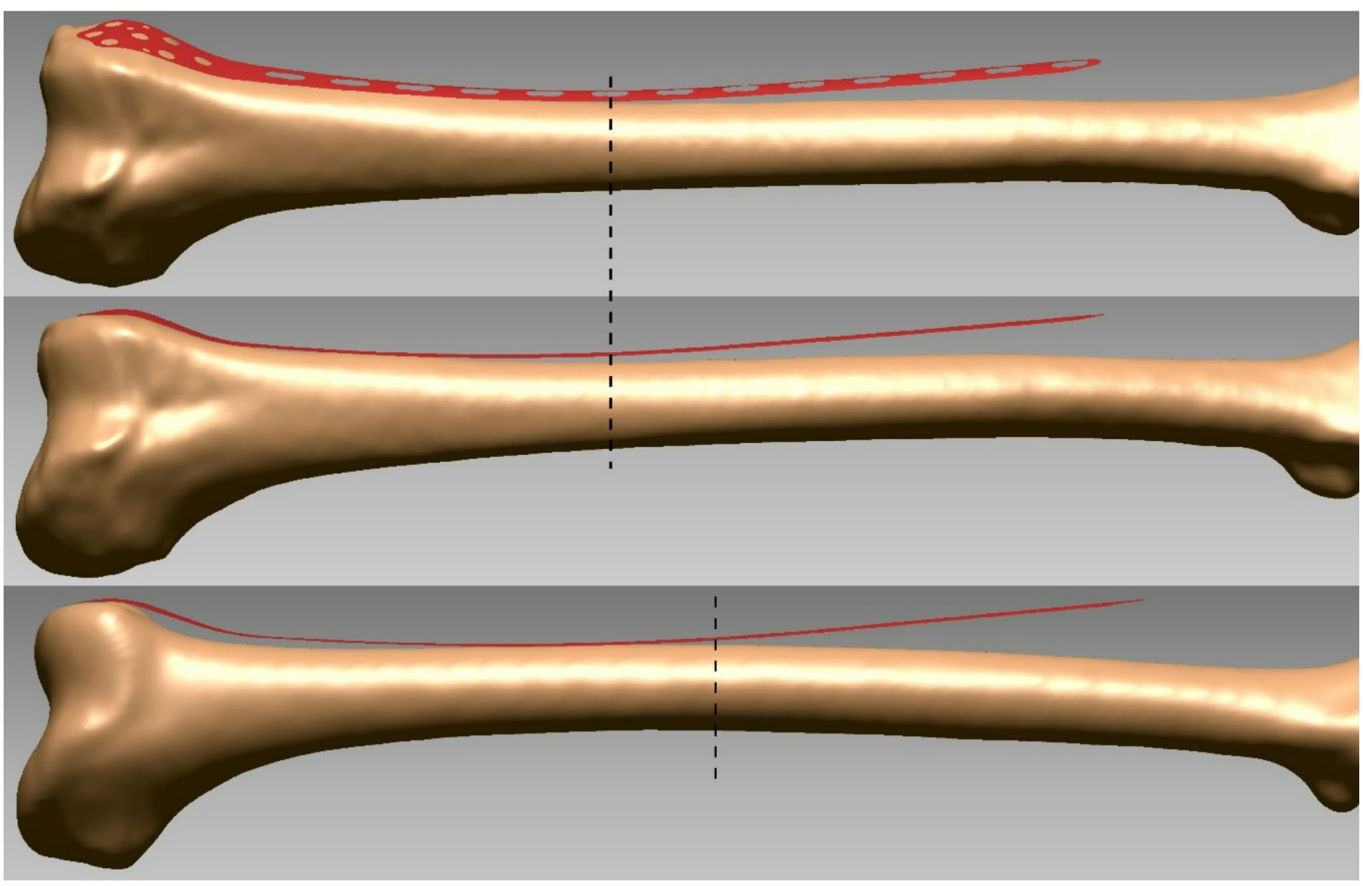
Examples of plate fit for femora representing average patients. (**Top**) Caucasian 28-year old male, height = 173 cm, ROC = 935 mm, 13-hole plate, proximal tip plate-bone distance = 13.0 mm (shown in true AP view). (**Middle**) The above Caucasian femur externally rotated to visualise the lateral plate profile. (**Bottom**) Vietnamese 45-year old female, height = 152 cm, ROC = 862 mm, 11-hole plate, proximal tip plate-bone distance = 15.8 mm. Screw hole 6 is indicated by the vertical dashed lines on all bones

For Caucasian femora, only 11-hole (*n* = 34) and 13-hole (*n* = 46) plates were used, while 9- (*n* = 4), 11- (*n* = 55), and 13-hole (*n* = 20) were used for Vietnamese. Satisfactory plate conformity was achieved from the plate head up to screw hole 6, with most measurement locations fitting between 52 and 100% of bones for both ethnicities (Fig. 3). The exceptions were locations (Caucasian: 3rd to 6th point, Vietnamese: 2nd to 7th point) along the posterior plate edge in the metaphyseal to distal shaft region, with plate fit of 5–50%. The most anterior point on the plate head also fitted poorer (*p* = 0.001) for Caucasians (45%) compared to Vietnamese (65%), although the most posterior point fit 100% for both.

Other ethnicity specific differences were: a larger (*p* < 0.001) mean plate-bone distance at the plate tip of 1.8 mm (0.7–5.9 mm) for Caucasian vs. 1.3 mm (0.3–2.5 mm) for Vietnamese; and smaller (*p* < 0.026) mean plate-bone distances along the 2nd and 3rd rows of distal measurement points ranging from 1.0 to 3.4 mm for Caucasian vs. 1.4–4.3 mm for Vietnamese.

Around the meta- to diaphyseal transition (4th row of distal measurement points), mean plate-bone distances ranged from 1.4 mm (anterior) to 3.8 mm (posterior) for Caucasian, and 1.4 to 4.1 mm for Vietnamese femora. Maximum distances ranged from 4.3 to 7.4 mm for Caucasian and 3.9–9.2 mm for Vietnamese, with no significant differences between the ethnicities.

For both ethnicities, there was proximal plate misfit from hole 8 onwards, with fit ranging from 0 to 41% in this region, and a mean distance of 11.6 mm (1–27 mm) at the proximal plate tip for Caucasian and 16.3 mm (4.8–45.5 mm) for Vietnamese femora. Plate misfit was worse (*p* ≤ 0.017) for Vietnamese femora in this region (Fig. 3).

For the entire dataset, older subjects had larger plate-bone distances for Criteria 1 and 2 but not the third (Crit. 1: 1.08 mm vs. 0.79 mm, *p* = 0.014; Crit. 2: 1.67 mm vs. 1.42 mm, *p* = 0.013; Crit. 3: 3.43 mm vs. 3.14 mm, *p* = 0.226). Although the height difference was marginally non-significant (*p* = 0.051), older patients had a smaller (861 mm vs. 906 mm, *p* = 0.030) ROC. For Caucasian femora, age had no impact on height and ROC differences (Tables 1 and 2), and limited impact on plate fit apart from larger plate-bone distances at the distal most anterior point (Criteria 1) (Tables 3 and 4). Older Vietnamese, however, were of shorter height (*p* = 0.043), had a smaller (*p* < 0.001) ROC, with larger plate-bone distances for most of the plate compared to younger subjects (Tables 1, 2, 3 and 4).

Sex affected plate fit for the entire dataset, as females had larger (*p* < 0.001) plate-bone distances (Crit. 3) for most of the shaft; they were also of shorter height (*p* < 0.001) and had a smaller (*p* = 0.003) ROC. Female Caucasians were on average of shorter height (*p* < 0.001), had a smaller (*p* = 0.048) ROC, and their plate-bone distances for most of the shaft (Crit. 3) were larger (*p* = 0.035). Similarly, Vietnamese female had larger (*p* < 0.001) plate-bone distances for most of the shaft (Crit. 3), were of shorter height (*p* < 0.001), and had a smaller (*p* < 0.001) ROC (Tables 1, 2, 3 and 4).

Overall, based on the three defined criteria, anatomical fitting was not achieved for any bones in our dataset (Table 3).

A plate ROC = 940 mm was obtained from the reverse engineered plate. The median ROC of 976 mm (615–1739 mm) for Caucasians was larger (*p* < 0.001) than the 846 mm (471–1505 mm) for Vietnamese. Caucasians were also taller (*p* < 0.035) overall and amongst the age subgroups, except for old males (*p* = 0.16). For the combined ethnicities, the median femoral ROC was 897 mm.

For the entire dataset, ROC positively correlated (r_s_=0.45; *p* < 0.001) with height. Height and ROC positively correlated (r_s_=0.44, 0.36; *p* < 0.001) with the distal plate tip-bone distance, while most plate-bone distances in the proximal metaphysis and in the shaft exhibited negative correlations (r_s_=-0.17 to -0.55; *p* < 0.035) (Table 1 Supplement). Age did not correlate with height or ROC, and only showed positive association (r_s_=0.23 to 0.24; *p* < 0.005) with plate-bone distances along the 1st row of distal measurement points.

**Table 1:** Descriptive statistics of data samples

Ethnicity	Young/Old	Sex	Age, median ± SD, (range), years	Height, median ± SD, (range), cm	ROC, median ± SD, (range), mm
Caucasian	Both	All (*n* = 80)	57 ± 25.6	164 ± 9.6	976 ± 226
		Male (*n* = 40)	57 ± 24.9 (25–96)	170 ± 9.3 (150–193)	984 ± 246 (633–1739)
		Female (*n* = 40)	58 ± 26.6 (21–96)	159 ± 6.7 (150–180)	976 ± 204 (615–1624)
	Young	All (*n* = 40)	31 ± 7.4	165 ± 10.4	938 ± 211
		Male (*n* = 20)	31 ± 6.9 (25–48)	171 ± 10.8 (150–193)	919 ± 206 (783–1462)
		Female (*n* = 20)	29 ± 7.6 (21–47)	160 ± 7.1 (152–180)	986 ± 218 (777–1624)
	Old (≥ 65)	All (*n* = 40)	81 ± 8.4	164 ± 8.8	1003 ± 243
		Male (*n* = 20)	81 ± 8.9 (65–96)	170 ± 7.8 (158–186)	1086 ± 274 (633–1739)
		Female (*n* = 20)	81 ± 8.1 (68–96)	158 ± 6.3 (150–173)	966 ± 184 (615–1360)
Vietnamese	Both	All (*n* = 79)	65 ± 13.8	158 ± 7.6	846 ± 174
		Male (*n* = 34)	62 ± 13.8 (31–82)	166 ± 5.3 (154–176)	912 ± 180 (533–1505)
		Female (*n* = 45)	67 ± 14.1 (33–84)	155 ± 4.7 (144–165)	804 ± 151 (471–1235)
	Young	All (*n* = 36)	47 ± 9.7	160 ± 7.2	898 ± 171
		Male (*n* = 19)	53 ± 10.4 (31–63)	167 ± 6.3 (154–176)	956 ± 180 (694–1505)
		Female (*n* = 17)	44 ± 7.9 (33–60)	158 ± 4.4 (149–165)	862 ± 142 (675–1235)
	Old (≥ 65)	All (*n* = 43)	68 ± 5.1	155 ± 7.6	791 ± 156
		Male (*n* = 15)	70 ± 5.2 (65–82)	165 ± 4.0 (156–171)	907 ± 161 (533–1216)
		Female (*n* = 28)	68 ± 5.0 (65–84)	153 ± 4.5 (144–165)	781 ± 146 (471–1159)

**Table 2:** Statistical significance (Mann-Whitney U) tests of descriptive sample data (significant values appear in bold)

Groups compared	Age, *p*-value	Height, *p*-value	ROC, *p*-value
Caucasian males vs. females (all)	0.53	**< 0.001**	0.43
Caucasian males vs. females (young)	0.10	**0.001**	0.29
Caucasian males vs. females (old)	0.90	**< 0.001**	**0.048**
Caucasian young vs. old (all)	**< 0.001**	0.41	0.77
Caucasian young vs. old (male)	**< 0.001**	0.47	0.16
Caucasian young vs. old (female)	**< 0.001**	0.29	0.22
Vietnamese males vs. females (all)	0.69	**< 0.001**	**< 0.001**
Vietnamese males vs. females (young)	0.063	**< 0.001**	**0.021**
Vietnamese males vs. females (old)	0.31	**< 0.001**	**0.044**
Vietnamese young vs. old (all)	**< 0.001**	**0.043**	**< 0.001**
Vietnamese young vs. old (male)	**< 0.001**	0.73	**0.025**
Vietnamese young vs. old (female)	**< 0.001**	**0.029**	**0.020**
Caucasian vs. Vietnamese (all)	0.77	**< 0.001**	**< 0.001**
Caucasian vs. Vietnamese (all males)	0.84	**0.013**	0.072
Caucasian vs. Vietnamese (all females)	0.87	**< 0.001**	**< 0.001**
Caucasian vs. Vietnamese (all young)	**< 0.001**	**0.020**	0.15
Caucasian vs. Vietnamese (all old)	**< 0.001**	**< 0.001**	**< 0.001**
Caucasian vs. Vietnamese (young males)	**< 0.001**	**0.034**	0.55
Caucasian vs. Vietnamese (young females)	**< 0.001**	**0.019**	**0.018**
Caucasian vs. Vietnamese (old males)	**0.002**	0.16	**0.005**
Caucasian vs. Vietnamese (old females)	**< 0.001**	**< 0.001**	**0.001**

**Table 3:** Description of (1) plate-bone distances and (2) percentages of bones satisfying fit criteria, by ethnicity, age and sex

Ethnicity	Young/Old	Sex	Criteria 1*		Criteria 2†		Criteria 3‡		All criteria
			Plate-bone distance Median ± SD, (range), mm	Satisfied criteria *n*, (%)	Plate-bone distance Median ± SD, (range), mm	Satisfied criteria *n*, (%)	Plate-bone distance Median ± SD, (range), mm	Satisfied criteria *n*, (%)	Satisfied criteria *n*, (%)
Caucasian	Both	All (*n* = 80)	1.10 ± 1.04	36 (45.00)	1.41 ± 0.51	4 (5.00)	3.12 ± 1.92	1 (1.25)	0 (0.00)
		Male (*n* = 40)	1.11 ± 1.29 (0.54–8.47)	18 (45.00)	1.39 ± 0.53 (0.12–6.99)	3 (7.50)	2.97 ± 1.72 (0.08–23.07)	1 (2.50)	0 (0.00)
		Female (*n* = 40)	1.10 ± 0.71 (0.24–4.27)	18 (45.00)	1.42 ± 0.48 (0.17–6.69)	1 (2.50)	3.47 ± 2.01 (0.10-27.07)	0 (0.00)	0 (0.00)
	Young	All (*n* = 40)	0.78 ± 1.32	26 (65.00)	1.37 ± 0.64	3 (7.50)	3.14 ± 2.06	1 (2.50)	0 (0.00)
		Male (*n* = 20)	0.80 ± 1.76 (0.54–8.47)	12 (60.00)	1.37 ± 0.71 (0.12–6.99)	2 (10.00)	2.99 ± 1.85 (0.08–23.07)	1 (5.00)	0 (0.00)
		Female (*n* = 20)	0.72 ± 0.63 (0.24–2.44)	14 (70.00)	1.34 ± 0.58 (0.24–6.69)	1 (5.00)	3.47 ± 2.21 (0.10-27.07)	0 (0.00)	0 (0.00)
	Old (≥ 65)	All (*n* = 40)	1.26 ± 0.65	10 (25.00)	1.46 ± 0.34	1 (2.50)	3.08 ± 1.79	0 (0.00)	0 (0.00)
		Male (*n* = 20)	1.28 ± 0.56 (0.70–3.03)	6 (30.00)	1.46 ± 0.28 (0.23–5.57)	1 (5.00)	2.91 ± 1.62 (0.28–21.62)	0 (0.00)	0 (0.00)
		Female (*n* = 20)	1.26 ± 0.74 (0.65–4.27)	4 (20.00)	1.51 ± 0.38 (0.17–5.88)	0 (0.00)	3.57 ± 1.83 (0.30-20.82)	0 (0.00)	0 (0.00)
Vietnamese	Both	All (*n* = 79)	0.79 ± 0.43	51 (64.56)	1.79 ± 0.68	3 (3.80)	3.49 ± 2.40	0 (0.00)	0 (0.00)
		Male (*n* = 34)	0.79 ± 0.35 (0.2–1.77)	26 (76.47)	1.63 ± 0.60 (0.15–7.86)	2 (5.88)	2.85 ± 1.41 (0.20-20.54)	0 (0.00)	0 (0.00)
		Female (*n* = 45)	0.83 ± 0.47 (0.13–2.30)	25 (55.56)	2.01 ± 0.69 (0.13–8.60)	1 (2.22)	3.87 ± 2.70 (0.08–45.46)	0 (0.00)	0 (0.00)
	Young	All (*n* = 36)	0.80 ± 0.34	24 (66.67)	1.63 ± 0.53	2 (5.56)	3.18 ± 1.64	0 (0.00)	0 (0.00)
		Male (*n* = 19)	0.79 ± 0.32 (0.3–1.66)	14 (73.68)	1.56 ± 0.55 (0.15–6.23)	1 (5.26)	2.69 ± 1.29 (0.20-20.34)	0 (0.00)	0 (0.00)
		Female (*n* = 17)	0.83 ± 0.36 (0.29–1.81)	10 (58.82)	1.73 ± 0.51 (0.13–5.50)	1 (5.88)	3.64 ± 1.80 (0.08–23.27)	0 (0.00)	0 (0.00)
	Old (≥ 65)	All (*n* = 43)	0.79 ± 0.49	27 (62.79)	2.09 ± 0.74	1 (2.33)	3.86 ± 2.77	0 (0.00)	0 (0.00)
		Male (*n* = 15)	0.79 ± 0.40 (0.20–1.77)	12 (80.00)	1.75 ± 0.68 (0.24–7.86)	1 (6.67)	3.16 ± 1.54 (0.25–20.54)	0 (0.00)	0 (0.00)
		Female (*n* = 28)	0.80 ± 0.53 (0.13–2.30)	15 (53.57)	2.45 ± 0.72 (0.18–8.60)	0 (0.00)	4.30 ± 3.05 (0.28–45.46)	0 (0.00)	0 (0.00)

*Criteria 1: Plate to bone distance ≤ 1 mm at most anterior distal location; Number of measurements involved in calculating mean (SD) = 1

†Criteria 2: Plate to bone distance ≤ 2 mm at nine points over the metaphyseal region, including distal tip; Number of measurements involved in calculating mean (SD) = 9

‡Criteria 3: Plate to bone distance ≤ 3 mm for points (anterior, centre, posterior) along the shaft spaced at ~ 4 cm intervals, including proximal tip (Number of measurements involved in calculating mean (SD) = 16 (9 holes), 19 (11 holes), 22 (13 holes))

**Table 4:** Differences˄ in (1) mean plate-bone distances and (2) percentages of bones satisfying fit criteria, by ethnicity, age and sex (significant values appear in bold)

Groups compared	Criteria 1*		Criteria 2†		Criteria 3‡	
	Plate-bone distance, *p*-value	Satisfied criteria *p*-value	Plate-bone distance, *p*-value	Satisfied criteria *p*-value	Plate-bone distance, *p*-value	Satisfied criteria *p*-value
Caucasian males vs. females (all)	0.593	1.000	0.836	0.615	**0.035**	1.000
Caucasian males vs. females (young)	0.234	0.507	0.978	1.000	0.218	1.000
Caucasian males vs. females (old)	0.914	0.465	0.829	1.000	0.083	1.000
Caucasian young vs. old (all)	**< 0.001**	**< 0.001**	0.142	0.615	0.456	1.000
Caucasian young vs. old (male)	**0.024**	0.057	0.317	1.000	0.409	1.000
Caucasian young vs. old (female)	**0.013**	**0.001**	0.223	1.000	0.808	1.000
Vietnamese males vs. females (all)	0.237	0.054	0.053	0.574	**< 0.001**	1.000
Vietnamese males vs. females (young)	0.495	0.345	0.634	1.000	**0.013**	1.000
Vietnamese males vs. females (old)	0.320	0.087	0.090	0.349	**0.018**	1.000
Vietnamese young vs. old (all)	0.914	0.720	**0.019**	0.589	**0.018**	1.000
Vietnamese young vs. old (male)	0.627	1.000	0.555	1.000	0.199	1.000
Vietnamese young vs. old (female)	0.870	0.731	**0.029**	0.378	0.120	1.000
Caucasian vs. Vietnamese (all)	**0.001**	**0.013**	**< 0.001**	1.000	0.099	1.000
Caucasian vs. Vietnamese (all males)	**0.002**	**0.006**	0.163	1.000	0.708	1.000
Caucasian vs. Vietnamese (all females)	0.158	0.331	**< 0.001**	1.000	0.151	1.000
Caucasian vs. Vietnamese (all young)	0.847	0.878	0.066	1.000	0.823	1.000
Caucasian vs. Vietnamese (all old)	**< 0.001**	**< 0.001**	**0.002**	1.000	**0.019**	1.000
Caucasian vs. Vietnamese (young males)	0.368	0.365	0.227	1.000	0.546	1.000
Caucasian vs. Vietnamese (young females)	0.594	0.478	0.190	1.000	0.831	1.000
Caucasian vs. Vietnamese (old males)	**0.001**	**0.003**	0.395	1.000	0.224	1.000
Caucasian vs. Vietnamese (old females)	**0.025**	**0.019**	**0.002**	1.000	0.107	1.000

˄Statistical tests used: Mann Whitney U test for comparing the difference in means between two groups, Chi-Square test for comparing the difference in satisfying plate fit criteria between two groups

*Criteria 1: Plate to bone distance ≤ 1 mm at most anterior distal location; Number of measurements involved in calculating mean (SD) = 1

†Criteria 2: Plate to bone distance ≤ 2 mm at nine points over the metaphyseal region, including distal tip; Number of measurements involved in calculating mean (SD) = 9

‡Criteria 3: Plate to bone distance ≤ 3 mm for points (anterior, centre, posterior) along the shaft spaced at ~ 4 cm intervals, including proximal tip (Number of measurements involved in calculating mean (SD) = 16 (9 holes), 19 (11 holes), 22 (13 holes))

## Discussion

While this study demonstrated satisfactory fit from plate head to midshaft for both ethnicities, there was proximal plate misfit from hole 8 onwards. The Caucasian mean plate-bone distance of 11.6 mm at the proximal tip was smaller (*p* ≤ 0.017) than the mean 16.3 mm misfit on the Vietnamese femora.

To our knowledge, this is the first report of proximal shaft plate misfit for Caucasians, which surprisingly was of similar magnitude to the 11.4 mm reported for the same plate fixed to Korean cadaver bones [15]. However, since a longer plate is more difficult to fit, using a 13-hole plate for 58% of our Caucasians and for 25% of our Vietnamese bones likely contributed to larger proximal plate tip to bone distances in our study compared to the Korean study [15], which used an 11-hole plate for all bones. Although the results confirm our hypothesis of better proximal plate fit for Caucasians than Vietnamese, due to plate length bias in favour of the Korean study, they do not allow us to generalise the findings to Asians in general.

Although, the mean plate-bone distances in the subcondylar region (2nd and 3rd rows of distal measurement points) were smaller (*p* < 0.026) for Caucasian (1.0–3.4 mm) compared to Vietnamese femora (1.4–4.3 mm), around the meta- to diaphyseal transition (4th row of measurement points) there was no significant difference. Overall, the mean metaphyseal plate-bone-distance (1.4–4.1 mm) for both ethnicities in our study was below the 6.6 mm mean (range 5.5–7.5 mm) reported for the same plate in pre-TKA patients [5]. Further, our results are also below the 5.5–9.2 mm range of mean distances on normal femora reported for pre-contoured distal femur plates from other major manufacturers [2]. Some of these differences might be due to differences in plate designs, while others may be explained by the differences between 3D modelling in our study versus 2D templating of radiographs by Bedard et al. [2] and Campbell et al. [5]. In our study, we positioned the plate to be flush with the surface of the lateral condyle, particularly at the most anterior distal plate tip, to limit plate protrusion close to the iliotibial band. Due to the 10° medial angulation [7] of the lateral condyle surface, the plate undersurface in our study also appeared rotated in the lateral view (see Fig. 3), whereas with templating, a plate lateral cross-section profile is fitted to the femur lateral profile. In addition, anatomical differences might exist between normal femoral morphology and that of TKA patients [5, 35].

The Caucasian median ROC (976 mm) of our sample is in the range of reported values (963–1523 mm) [12, 21, 27, 30], with no differences (*p* = 0.43, *p* = 0.77) between male (984 mm) and female (976 mm), nor between young (938 mm) and old (1003 mm). In comparison, the obtained Vietnamese median ROC (846 mm) is smaller (*p* < 0.001), with differences (*p* < 0.001) between both male (912 mm) and female (804 mm), and young (898 mm) vs. old (791 mm). To our knowledge, we are the first to report ROC values for Vietnamese femora. Both Caucasian and Vietnamese median ROC are within one standard deviation of the 940 mm plate ROC, with the latter deviating < 1% from the mean ROC of the ethnicities combined. This suggests that the plate ROC is an appropriate compromise for the treatment of an ethnically diverse patient population.

The median height of our Caucasian male (170 cm) sample is slightly below the mean (175–176 cm) of Australian males, while our female mean (159 cm) is close to the Australian female mean (162 cm) [22, 29]. In comparison, our Vietnamese males (166 cm) and females (155 cm) were significantly (*p* < 0.014) shorter, but in the range of reported Vietnamese male (161–166 cm) and female (152–155 cm) mean heights [4, 11, 13, 18, 31]. While there was no height difference between our young and old Caucasian samples, our older Vietnamese female cohort was shorter (*p* = 0.029) compared to the young, but the male cohort was not.

Our data displayed a moderate positive correlation (r_s_=0.45; *p* < 0.001) between height and ROC, which is slightly higher than previous reported values (*r* = 0.36 to 0.39) [21, 27, 30]. While a weak age-ROC association has been reported [16, 30], our data did not support that and is on par with Karakas et al. [16] who found no correlation for males. As age did not correlate with ROC, it is likely that a difference in mean height was the cause for the observed ROC differences between the sexes, age groups, and ethnicities. This is supported by Thiesen et al. [30], who reported no ROC difference between age and length matched Asian and Caucasian femora.

Furthermore, while both height and ROC negatively correlated with most plate-bone distances in the shaft, due to the positive height-ROC correlation, it is likely height difference was the main driver of the poorer plate fit observed for Vietnamese and females who both were shorter compared to Caucasians and males. This also aligns with the observed mismatch between bone and plate ROC, which was more pronounced for Vietnamese femora, females in particular.

While CT imaging is the gold standard for 3D bone modelling, work by Rathnayaka et al. [24, 25] has demonstrated that MRI based 3D bone models are of comparable accuracy. Since plate fit in this study was assessed in the range of millimetres, while MRI based 3D model accuracy, including surface smoothing, is in the sub-voxel range of 0.32 ± 0.02 mm [24], we do not expect that the imaging modality will have any important effects on the results and findings obtained. A limitation of our study is that we have assessed the fitting of only one plate type and therefore it is unknown to what extent our findings can be generalised. Furthermore, we have not considered any intra-operative contouring of the plate with the aid of bending irons, for reducing proximal shaft-plate misfit [5, 15]. A strength of our study is that plate fit was assessed for an age and ethnically diverse dataset. To our knowledge this is the first study to report on the effect of femoral bowing on plate fit.

## Conclusions

Of the assessed patient variables, sex, height, ethnicity, and femoral bowing all had significant impacts on plate fit, while the effect of age was limited. However, due to sex and ethnicity related height differences, our data suggests that patient height and femoral bowing are the main variables affecting fit. The observed proximal plate misfit for Caucasians, similar in magnitude to Koreans, indicates that the plate might exhibit less anatomical conformity than is generally assumed. For cases where the plate is used as a reduction tool, pre-operative templating of the intact contralateral femur can potentially identify any plate misfit, in particular, for short stature patients and/or patients with strong anterior femoral bowing.

## Supplementary Information

Below is the link to the electronic supplementary material.

Supplementary file1 (DOCX 20 KB)

## Data Availability

No datasets were generated or analysed during the current study.
