# Supplementary material for: Effect of age, sex, height, ethnicity, and femoral bowing on the anatomical fitting of the LCP distal femur plate
Source: Arch Orthop Trauma Surg. 2025 Oct 14;145(1):472. doi: 10.1007/s00402-025-06079-1 (PMC12521290; doi:10.1007/s00402-025-06079-1)
Supplement: Supplementary file 1 — Supplementary file1 (DOCX 20 KB) [file 402_2025_6079_MOESM1_ESM.docx]

**Table 1-Supplement**. Correlation tests (Spearman) for age, height, ROC, and plate-bone distance measurements at locations of points shown in Figure 2.

| **Parameter** | **DistTip**  **(p value)** | **Dist1Ant** | **Dist1Cent** | **Dist1Post** | **Dist2Ant** | **Dist2Cent** | **Dist2Post** | **Dist3Ant** | **Dist3Cent** | **Dist3Post** |
| --- | --- | --- | --- | --- | --- | --- | --- | --- | --- | --- |
| Age | -0.013 (0.88) | 0.237 (0.003) | 0.230 (0.004) | 0.228 (0.004) | 0.146 (0.067) | 0.083 (0.299) | -0.039 (0.624) | 0.161 (0.043) | -0.015 (0.855) | -0.046 (0.561) |
| Height | 0.439 (<0.001) | 0.154 (0.052) | 0.069 (0.39) | 0.031 (0.70) | -0.321 (<0.001) | -0.300 (<0.001) | -0.414 (<0.001) | -0.397 (<0.001) | -0.329 (<0.001) | -0.467 (<0.001) |
| ROC | 0.364 (<0.001) | -0.027 (0.74) | -0.114 (0.15) | 0.147 (0.065) | -0.243 (0.002) | -0.254 (0.001) | -0.168 (0.034) | -0.360 (<0.001) | -0.337 (<0.001) | -0.313 (<0.001) |

| **Parameter** | **Shaft1Ant** | **Shaft1Cent** | **Shaft1Post** | **Shaft2Ant** | **Shaft2Cent** | **Shaft2Post** | **Shaft3Ant** | **Shaft3Cent** | **Shaft3Post** |
| --- | --- | --- | --- | --- | --- | --- | --- | --- | --- |
| Age | 0.122 (0.125) | -0.075 (0.345) | -0.136 (0.087) | 0.041 (0.607) | 0.042 (0.559) | 0.013 (0.873) | 0.105 (0.189) | 0.111 (0.163) | 0.057 (0.475) |
| Height | 0.105 (0.189) | 0.013 (0.870) | -0.250 (0.001) | 0.098 (0.219) | -0.202 (0.011) | -0.394 (<0.001) | 0.097 (0.225) | -0.193 (0.015) | -0.457 (<0.001) |
| ROC | -0.038 (0.632) | -0.180 (0.023) | -0.385 (<0.001) | 0.124 (0.119) | -0.326 (<0.001) | -0.536 (<0.001) | 0.254 (0.001) | -0.317 (<0.001) | -0.552 (<0.001) |

| **Parameter** | **Shaft4Ant** | **Shaft4Cent** | **Shaft4Post** | **Shaft5Ant** | **Shaft5Cent** | **Shaft5Post** | **Shaft6Ant** | **Shaft6Cent** | **Shaft6Post** |
| --- | --- | --- | --- | --- | --- | --- | --- | --- | --- |
| Age | 0.087 (0.274) | 0.072 (0.365) | 0.030 (0.703) | 0.069 (0.389) | 0.040 (0.618) | 0.007 (0.930) | 0.050 (0.535) | 0.042 (0.603) | 0.031 (0.704) |
| Height | -0.188 (0.018) | -0.252 (0.001) | -0.389 (<0.001) | -0.448 (<0.001) | -0.444 (<0.001) | -0.432 (<0.001) | -0.546 (<0.001) | -0.517 (<0.001) | -0.473 (<0.001) |
| ROC | 0.008 (0.919) | -0.269 (<0.001) | -0.461 (<0.001) | -0.408 (<0.001) | -0.401 (<0.001) | -0.369 (<0.001) | -0.479 (<0.001) | -0.479 (<0.001) | -0.455 (<0.001) |

| **Parameter** | **Shaft7Ant** | **Shaft7Cent** | **Shaft7Post** | **ProxTip** |
| --- | --- | --- | --- | --- |
| Age | -0.012 (0.923) | -0.007 (0.953) | -0.005 (0.971) | 0.074 (0.355) |
| Height | -0.415 (<0.001) | -0.367 (0.002) | -0.328 (0.007) | -0.340 (<0.001) |
| ROC | -0.489 (<0.001) | -0.471 (<0.001) | -0.442 (<0.001) | -0.457 (<0.001) |
